# Respiratory Muscle Function in Older Adults with Chronic Respiratory Diseases after Pulmonary Rehabilitation in Subterranean Salt Chambers

**DOI:** 10.3390/jcm12155120

**Published:** 2023-08-04

**Authors:** Sylwia Mętel, Magdalena Kostrzon, Justyna Adamiak, Paweł Janus

**Affiliations:** 1 Department of Motor Rehabilitation, Institute of Applied Sciences, University of Physical Education in Krakow, 31-571 Krakow, Poland; justyna.adamiak@awf.krakow.pl; 2 ‘Wieliczka’ Salt Mine Health Resort, 32-020 Wieliczka, Poland; mkostrzon@interia.pl (M.K.); pawel.wojciech.janus@gmail.com (P.J.)

**Keywords:** the elderly, speleotherapy, subterraneotherapy, maximum inspiratory pressure, maximum expiratory pressure, sniff nasal inspiratory pressure, diaphragm

## Abstract

Training the respiratory muscles is a crucial aspect of pulmonary rehabilitation. The purpose of this study was to assess the function of respiratory muscles in older adults both before and after a period of pulmonary rehabilitation and treatment stay within the underground chambers of a salt mine. A total of 50 patients aged 65 years and older with chronic respiratory conditions was enrolled in the study. These participants underwent a 3-week subterranean pulmonary rehabilitation (PR) program in the “Wieliczka” Salt Mine. Levels of sniff nasal inspiratory pressure (SNIP), maximum inspiratory pressure (MIP), and maximum expiratory pressure (MEP) were measured using the MicroRPM both before and after the outpatient PR program conducted 135 m underground. A total of 44 patients with a mean age of 68.8 ± 2.9 years who completed the PR program and tests were included in the analysis. The average changes in the parameters of pulmonary function before and after the PR were: MIP 8.8 cmH_2_O, MEP 7.1 cmH_2_O, and SNIP 11.2 cmH_2_O (for *p* < 0.05). For patients older than 70 years, beneficial changes were only observed for MEP, which increased by 9.3 cmH_2_O (for *p* < 0.05). Speleotherapy combined with pulmonary rehabilitation improves respiratory muscle function in older adults with chronic respiratory diseases, mainly in terms of MEP. Therefore, a greater emphasis on inspiratory muscle training in the rehabilitation program should be considered.

## 1. Introduction

Respiratory muscles play a key role in respiratory function by generating the intrathoracic pressures that allow airflow, and they affect the shape and mobility of the chest, posture, and body balance. In addition, the diaphragm contributes to maintaining postural stability; its activity is important for the proper functioning of the heart and circulation in the lymphatic system and reduces the risk of developing chronic pain in the spine. Fatigue in the breathing muscles leads to an increase in the respiratory rate, which in turn limits exercise performance [1,2,3]. Along with the physiological aging of the human body, the respiratory muscles, classified as skeletal muscles, undergo age-related involution changes (senescence), as well as being subject to more specific factors that weaken their function. Several studies have indicated that compared to younger individuals, elderly people display greater levels of oxidative and nitrosative stress in their respiratory muscles [4]. In Janssens’s review, which investigated the physiological alterations in the respiratory system due to aging and their impact on respiratory mechanics, the author highlighted the gradual decrease in chest wall compliance, static elastic recoil of the lung, and strength of the respiratory muscles [5]. In people with chronic diseases of the respiratory tract, there is a secondary impairment of respiratory muscle function in both obstructive and restrictive diseases and, additionally, a weakening of their function due to the use of corticosteroid drugs, systemic inflammation and reduced physical activity in these patients [6]. Scientific reports indicate that for elderly patients, the goal of breathing exercises is to enhance lung function, strengthen respiratory muscles, and increase thoracoabdominal mobility. These exercises are deemed effective and safe as part of physical therapy [7]. 

Numerous studies have shown that respiratory muscle training (defined as repetitive and controlled exercises of moderate-to-high intensity) can improve the structural and functional properties of these muscles, in both healthy older individuals and COPD patients of a similar age [4,8,9,10]. Pulmonary rehabilitation (PR) improves a patient’s exercise tolerance and health-related quality of life and reduces limiting symptoms. While respiratory muscle training is a part of pulmonary rehabilitation, scientific evidence does not currently support the routine use of inspiratory muscle training as an essential component of this process [11]. 

In this context, speleotherapy emerges as a potential adjunctive treatment. Speleotherapy (subterranean therapy) is a unique form of climatotherapy that utilizes mine (primarily salt mine) or cave environments for the treatment of chronic respiratory diseases [12]. The environment in which pulmonary rehabilitation is conducted plays a significant role in the recovery process. Programs carried out in locations with low pollution levels, such as pollution-free underground salt chambers or caves, yield substantial health improvements. This environment thus complements and enhances the effects of respiratory muscle training in pulmonary rehabilitation [13,14,15]. The specific microclimate is characterized by an absence of pollutants and allergens, a high concentration of salt aerosol, high relative humidity, stable temperature, elevated atmospheric pressure, ionization, and deprivation of external stimuli. Both the climatic conditions and the unique scenery of the underground salt chambers adapted for the PR program encourage older individuals with chronic respiratory diseases to actively participate in respiratory training sessions [16,17,18]. 

Maximal inspiratory pressure (MIP) and maximal expiratory pressure (MEP) are straightforward, user-friendly, and non-invasive measurements of respiratory muscle strength at the mouth. However, definitive standards for these indices have not yet been firmly established [19]. The values of MIP and MEP are affected by the methodology used for measurement, the individual’s motivation, as well as their gender and age [20]. These tests broadly assess the neuromuscular function of the combined diaphragm, abdominal, intercostal, and accessory muscles. Sometimes, a sniff nasal inspiratory pressure (SNIP) is utilized in place of MIP. The study conducted by Terzi et al. [21] demonstrated that SNIP is also more consistent than MIP in its results. 

From the literature review, it seems that there is a scarcity of research on the use of speleotherapy in pulmonary rehabilitation for older adults. In our previous article regarding this project, which involved participants aged 65 and older with chronic respiratory conditions, we described the impact of combining speleotherapy with pulmonary rehabilitation on the dynamic balance and chest mobility of older adults [18]. Given that the rehabilitation and treatment program at the “Wieliczka” Salt Mine Health Resort also includes walking and aerobic training, in this article we would like to present further results of this project pertaining to measurements of respiratory muscle function using MIP, MEP, and SNIP.

### Aim of the Study

The goal of the study was to assess the respiratory muscle function of older adults before and after their rehabilitation and treatment stay in the underground salt mine chambers. 

## 2. Materials and Methods

### 2.1. Study Design

This was a prospective study of chronic respiratory diseases conducted at the “Wieliczka” Salt Mine Health Resort in Wieliczka, Poland from June 2017 to December 2019. 

### 2.2. Participants

The exclusion criteria for treatment in the underground salt chambers were medical contraindications to subterraneotherapy and speleotherapy, such as:

- Active cancer or cancer that has not been in remission for at least five years
- Primary or secondary immunodeficiencies
- Infectious diseases during their active clinical, acute, and chronic phases (being a carrier is not a contraindication)
- Serious musculoskeletal damage that prevents independent movement
- Severe respiratory and circulatory failure limiting life activity to a resting lifestyle
- Increased intracranial pressure, for example due to infectious diseases or trauma
- Recent (up to 6 months) surgical procedures or trauma to the chest, abdominal cavity, craniomaxillofacial region, brain, or eye area
- Recent (up to 6 months) heart attack or stroke
- Aneurysms of cerebral vessels and aorta
- Severe heart valve defects
- Diagnosed arrhythmias that threaten loss of consciousness
- Uncontrolled hypertension, hemorrhagic disorders
- Mental disorders
- Claustrophobia
- Rheumatic diseases involving the skeletal system
- Epilepsy presenting as focal seizures with consciousness disturbances and focal seizures evolving to bilateral tonic–clonic seizures
- Pregnancy

The participants were all aged 65 years or older with chronic respiratory conditions. The inclusion criteria also included:

1. No contraindications for speleotherapy, as confirmed by a doctor during qualification.
2. A minimum score of 10 points on the short physical performance battery test, which assesses the risk of disability in older individuals [22,23].
3. A medical indication for pulmonary rehabilitation using subterraneotherapy methods (for chronic respiratory disease), confirmed by a doctor during qualification.
4. Written, informed consent to participate in the project.

### 2.3. Ethics

Information forms about the project were distributed among patients who were consulted by the medical staff about participating in the underground rehabilitation and treatment stay. The study was explained to the volunteer participants, and they were enrolled in the study after their written consent was obtained. The study was conducted in accordance with the Declaration of Helsinki and was approved by the Regional Medical Ethics Board of Physicians in Krakow, Poland (pol. Okręgowa Izba Lekarska w Krakowie) No. 40/KBL/OIL/2018.

### 2.4. Intervention

Outpatient PR was conducted for a period of 3 weeks (6 h a day, for 5 days a week) 135 m underground at the “Wieliczka” Salt Mine Health Resort in Wieliczka, Poland. The daily schedule of the rehabilitation and treatment program included a supervised descent into the mine by medical staff (doctor, nurse, physiotherapist) using a shaft and a 700 m march on the uneven surface of the miners’ route. The program also included one session of group endurance training supervised by a physiotherapist using aerobic or resistance exercises for 30 min, one session of breathing exercises including posture control and respiratory muscle training with or without tools that give resistance during exhalation such as paper tubes, pinwheels, artificial feathers or purse-lip breathing for 30 min, and one session of specialized training using resistance tools including strength exercises or relaxation methods for 30 min. An educational panel was held three times a week, discussing methods of dyspnea control and behaviors during disease exacerbations, coping with chronic fatigue, sleep hygiene, healthy diet, and health-promoting behaviors. The panel was run by medical staff. The day concluded with a 700 m group walk to the shaft and ascent to the surface along the uneven surface of the miners’ route (Table 1).

### 2.5. Outcome Measures

**Figure 1:**
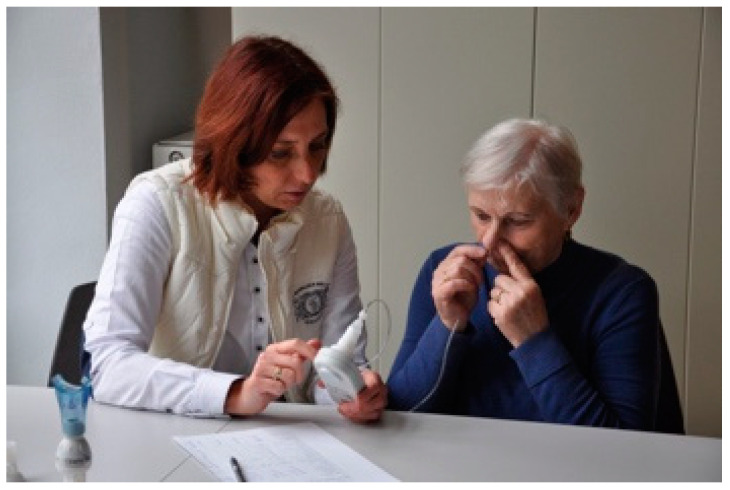
SNIP measurement.

Before and after the outpatient PR program conducted in underground salt chambers, the function of the respiratory muscles was assessed by measuring the maximum static pressures in the respiratory system. This measurement was performed using a portable, digital device known as MicroRPM [24]. The measurements of maximal respiratory pressures included MIP as an index of the strength of the diaphragm, and MEP as a measurement of the strength of abdominal and intercostal muscles. Results of the indirect assessment of respiratory muscles’ conditions were displayed on a digital readout panel. Specialized electronics calculated the pressure and presented the results in centimeters of water (cmH_2_O), with the measuring range being between −300 cmH_2_O and +300 cmH_2_O. Tests were carried out three times in a sitting position to establish the best value, according to the manufacturer’s instructions. MIP and MEP were measured after calibrating the measuring device, through the mouth, with a mouthpiece applied to the mouth and teeth. In order to assess MEP, the patient was asked to take the deepest possible breath, near total lung capacity (TLC) level, and then exhale maximally into the gauge for at least 2 s. The result displayed on the LCD screen represented the MEP averaged for 1 s. To properly perform the examination, it was checked whether there were any leaks in the connection system during the measurement, with the exception of the one in the gauge that prevents the generation of erroneous, high results by closing the glottis and compressing the air in the oral cavity using cheek muscles. In order to evaluate MIP, the patient was asked to exhale completely until the lungs were emptied to reach residual volume (RV), and then inhale as hard as possible through the mouthpiece for at least 2 s. The result displayed on the screen represented the maximum inspiratory pressure averaged for 1 s (Figure 1). The mouthpiece was sterilized after use. SNIP was also a parameter in testing the respiratory function of the elderly patients. The patient, with an inserted and tightly fitting nasal sensor, was asked and encouraged to exhale through the mouth until the lungs were completely emptied (RV), then close their mouth and inhale as strongly as possible through the nose. The SNIP test involved a short, sharp, voluntary inspiratory maneuver, measured through a plug occluding one nostril while the sniff was performed using the contralateral nostril. The test was carried out in a sitting position, with the use of three to five repetitions for each nostril. The plug was sterilized after use [24,25]. 

### 2.6. Statistical Analysis

The Student’s *t*-test for dependent samples was applied. Comparison of the values of quantitative variables in repeated measurements (before and after the rehabilitation program) was performed with the Student’s *t*-test for dependent samples, as the normal distribution of variables was confirmed by the Shapiro–Wilk test. The analyses were carried out using Statistica 13.3. A *p*-value ≤ 0.05 was considered significant.

## 3. Results

### 3.1. Sample Characteristic

**Figure 2:**
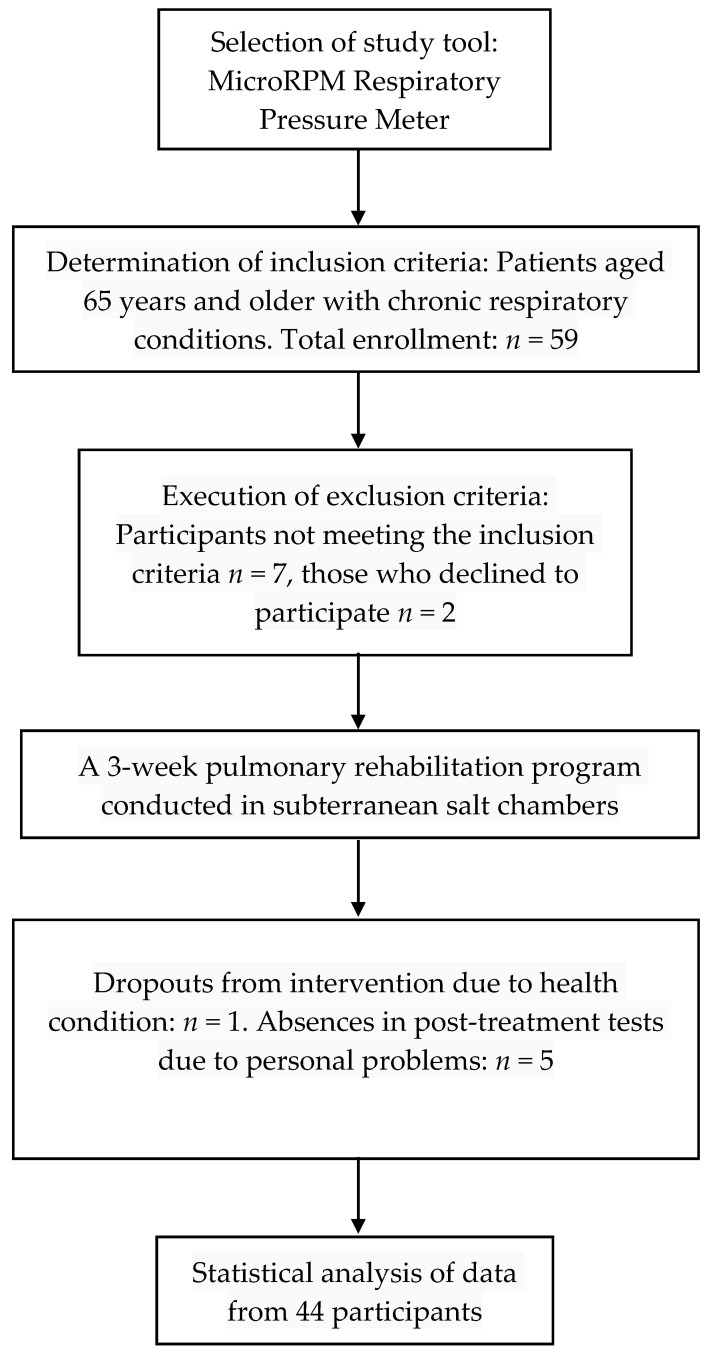
Flowchart for enrollment, allocation, and follow-up of participants.

As shown in Figure 2, out of 59 people screened for eligibility, 50 were recruited for a 3-week subterranean PR program in the “Wieliczka” Salt Mine Health Resort, according to predefined inclusion criteria. Some 44 participants with a mean age of 68.8 ± 2.9 and mean BMI of 28.5 ± 3.6, including 28 women with a mean age of 68.5 ± 3.2 years and mean BMI of 28.4 ± 3.8, and 16 men with a mean age of 69.4 ± 2.5 years and mean BMI of 28.6 ± 3.5, completed the intervention program and were therefore eligible for statistical analyses. Reasons for dropout included a deterioration in health due to influenza (*n* = 1), and absence in post-treatment tests due to personal problems (*n* = 5). A total of 26 patients (59% of the study group) had conditions of the lower respiratory tract such as bronchial asthma, COPD, and bronchiectasis, and 18 people (41% of the study group) had conditions of the upper respiratory tract such as sinusitis, pharyngitis, and laryngitis (Table 2). Back pain was declared by 16 women and 4 men (45% of patients), lower limb pain by 7 women and 2 men (20% of patients), and upper limb pain in 6 women and 3 men (20% of patients). A total of 14 women reported between one and ten falls and 6 men reported between one and three falls in the past 5 years.

### 3.2. Outcomes

Participants were assessed both before and after the 3-week intervention. When analyzing the results of respiratory muscle function for the entire group of older individuals studied, we observed a significant increase in mean values of MIP by 10.2%, MEP by 12.3%, and SNIP by 28.4% for *p* < 0.05 after the rehabilitation and treatment program. For patients with lower respiratory tract diseases, MEP increased by 10.7% and SNIP by 31.0%. Meanwhile, for those with upper respiratory tract diseases, MIP increased by 15.9% and MEP by 14.9% (Table 3). Taking into account the gender of all patients studied, a significant increase was observed after the PR program in the “Wieliczka” Salt Mine Health Resort. For women, the mean values for MIP increased by 14.1% and MEP by 11.3%; for men, MEP increased by 13.5% and SNIP by 45.5%. These results were statistically significant with a *p*-value less than 0.05 (Table 4). In the younger group of patients aged 65–69, significant improvements in respiratory muscle function were observed after the PR program in the underground therapeutic chambers. The average values of the measured parameters increased: MIP by 10.4%, MEP by 9.8%, and SNIP by 35.9%. However, in the older group of seniors aged 70 and over, improvements were observed only for MEP, which increased by 15.3%, with a *p*-value less than 0.05 indicating statistical significance (Table 5).

## 4. Discussion

The purpose of this study was to assess the function of respiratory muscles in older adults with chronic respiratory diseases both before and after a period of pulmonary rehabilitation (PR) and treatment stay within the underground chambers of a salt mine. The combination of speleotherapy and PR led to an increase in respiratory muscle function, most notably in terms of maximal expiratory pressure (MEP). 

Physical inactivity is a primary cause of most chronic diseases. The elderly in particular experience involutionary changes in skeletal muscles, including decreases in respiratory muscle strength. Therefore, it is advisable for them to participate in regular physical training that is adapted to their needs, preferably in a natural, pollution-free environment.

Pulmonary rehabilitation in the unique underground bioclimate of the “Wieliczka” Salt Mine Health Resort allows seniors with chronic respiratory diseases to participate in a physical exercise program. The program is supervised by a physiotherapist and is in line with the American Thoracic Society/European Respiratory Society recommendations. The types of activities include endurance training like walking, resistance exercises, and exercises that improve neuromuscular coordination, such as balance, coordination, and flexibility tasks. The program also features upper and lower limb training, relaxation exercises, and conscious efforts to improve posture and breathing quality [26]. In the context of breathing exercises, we primarily use techniques aimed at improving chest flexibility. These include exercises for respiratory and phonatory coordination, as well as exercises involving forced exhalation. Examples of the latter are exhaling through pursed lips or using small, disposable items like paper cups, straws, and feathers, or creating “plosive” sounds. However, inspiratory muscle training is conducted to a lesser extent. During the 3-week rehabilitation and treatment stay at the “Wieliczka” Salt Mine Health Resort, patients traverse a 700-m stretch of the mine gallery every day, from Monday to Friday. They descend via an elevator from the shaft to a depth of 135 m to reach the healing chambers, after which they walk the same distance back. This integral component of the pulmonary rehabilitation program, inclusive of walking training, has garnered significant attention due to its effectiveness in treating patients with respiratory diseases. This was exemplified in a blinded, randomized, and controlled clinical trial conducted by Farias et al. [27], where the effectiveness of an 8-week simple aerobic walking program was evaluated. The trial involved individuals aged between 40 and 85 years old diagnosed with chronic obstructive pulmonary disease (COPD). Noteworthy changes were identified upon the completion of the program in the trained group. The MicroRPM device was used to assess parameters related to respiratory function, revealing an increase of 11.9% in maximum expiratory pressure (MEP) and 1.9% in sniff nasal inspiratory pressure (SNIP). Further highlighted in the study were the considerable health improvements for patients with COPD, indicating that substantial advancements could be accomplished in a cost-effective manner [27]. 

The pulmonary rehabilitation program implemented in the undergrounds of the Wieliczka mine includes education on postural control during breathing, flexibility exercises, and maintaining the alignment of body segments. The diaphragm, as the primary inspiratory muscle, is responsible for 70–80% of ventilation in healthy individuals. It utilizes the pressure disparities between the thoracic and abdominal cavities for both respiration and maintaining postural stability, and it interacts with the other human body diaphragms: cranial, cervical, and pelvic floor. Additionally, it impacts fluid dynamics and posture [3,28,29]. Consequently, when improving respiratory function, even in individuals with chronic respiratory tract diseases, it is crucial to pay attention to postural control. This is due to the symbiotic relationship that exists between body posture and breathing [26].

A critical component of any rehabilitation program is evaluating the effectiveness of the therapeutic procedure. For patients with chronic respiratory diseases, in addition to conducting a functional assessment of the respiratory system (spirometry), it is beneficial to examine respiratory pressures [30]. Respiratory pressures should be considered as a global “output” of respiratory muscles, reflecting the strength of the muscles involved in the respiratory function, rather than solely as indicators of the muscles’ capacity to contract [31]. Maintaining equilibrium between the demand on respiratory muscles and their ability to function is of paramount significance. Intolerance to physical exertion due to respiratory muscle fatigue or shortness of breath triggers a vicious cycle. The patient halts physical activity, which subsequently leads to limitations in performing everyday tasks such as walking, reaching for objects, and getting dressed. Consequently, this results in reduced daily physical activity and a decline in their health-related quality of life. Performing tests that evaluate respiratory function, such as the assessment of respiratory muscle strength, effort tolerance tests like the 6 minute walk test or the Fullerton/Senior fitness test, allows for a more precise prescription of physical activity [26]. This includes determining the dose, frequency, and intensity of physical exercise for a given patient. In patients with poor exercise tolerance or respiratory muscle strength, it could be beneficial to segment physical activity into stages or to conduct it in intervals.

Our objective was to determine how the combined approach of pulmonary rehabilitation and subterraneotherapy influences the strength of respiratory muscles. In this study, we observed individuals aged 65 and above during a rehabilitation and treatment program conducted within the “Wieliczka” Salt Chambers. Our findings revealed a significant increase in the mean values of all measured respiratory pressures, as assessed by the MicroRPM meter. The least beneficial changes were found within the maximum inspiratory pressure (MIP) range. Further stratification by gender facilitated a comparison of our results with normative values established for healthy adults, as found in a systematic review with meta-analyses conducted by Sclauser Pessoa et al. [31]. We observed that the mean maximum inspiratory pressure (MIP) score for women at both baseline (72.5 cmH_2_O) and final (82.7 cmH_2_O) fell within the normal range for the patients’ age group (60–83 years, 57.8–82.9 cmH_2_O). Participation in the pulmonary rehabilitation program at the underground health resort in Wieliczka seemed to shift the result towards the upper limit of this range. Given the involutionary nature of changes in MIP over time, this increase in the mean MIP value in women can be deemed a favorable outcome. On the other hand, after the pulmonary rehabilitation program in the “Wieliczka” Salt Mine Health Resort, the average MIP value for men showed a slight increase from 110.9 cmH_2_O to 117.2 cmH_2_O. However, this difference was statistically insignificant. When juxtaposed with the established norms (the normal range for men aged 60–83 years is 66.1–100.8 cmH_2_O), it becomes apparent that the male participants in the study already exceeded the average MIP values during the initial measurement. This factor likely impeded the attainment of statistical significance due to the limited growth potential or the insufficient inclusion of inspiratory muscle training (IMT) in the rehabilitation program. The results demonstrated a significant improvement in maximum expiratory pressure (MEP) across all analyses conducted in the study. This could likely be attributed to the extensive utilization of expiratory muscle training (EMT) in the therapeutic procedures that were part of the pulmonary rehabilitation program implemented in the subterranean atmosphere of the “Wieliczka” Salt Mine. 

Patients with lower respiratory tract disease are typically known to exhibit lower respiratory muscle strength [26]. However, our study found that these patients demonstrated greater baseline muscle strength than those with upper respiratory tract disease. Mouth breathing, potentially resulting from upper airway blockage, may be associated with decreases in MIP and MEP values [32]. However, confirming this observation would necessitate a larger study group.

The sniff maneuver (SNIP) and maximum inspiratory pressure (MIP) are not interchangeable; rather, they are complementary. When both tests are performed, they can reduce the risk of false positives, thereby decreasing the incidence of inaccurately diagnosed respiratory muscle weakness by approximately 20% [33]. Uldry et al. [34] established reference values for the SNIP depending on age and sex in healthy individuals. For those aged 66–80 years, the reference values were above 91 cm H_2_O for elderly men and 75.5 cm H_2_O for elderly women. They concluded that SNIP was inversely correlated with age. However, Huang et al. [35], who studied 160 subjects with a mean age of 39.9 ± 16.5 (range 18–69 years) and divided them into different age and gender subgroups, concluded that sniff nasal inspiratory pressure (SNIP) does not decrease in elderly subjects. Analyzing the results of our study, we noted that both the average sniff nasal inspiratory pressure (SNIP) value for women at 39.5 cmH_2_O and for men at 39.3 cmH_2_O before the rehabilitation and treatment stay, as well as after its completion, with values for women at 47 cmH_2_O and for men at 57.2 cmH_2_O, were below the aforementioned reference values established for older individuals by Uldry et al. [34].

The beneficial and significant effect of inspiratory muscle training (IMT) using specialized equipment was observed in individuals with chronic lower respiratory tract diseases. In a randomized trial by Chung et al. [36], involving 60 adult patients with stable asthma aged 40–65 years, IMT using the Powerbreath IMT device over a 12-week period proved superior to conventional breathing exercises (BTE) in terms of increasing respiratory muscle strength. In a meta-analysis by Lötters et al. [37] regarding the effects of controlled inspiratory muscle training in patients with COPD, it was concluded that such training significantly improves inspiratory muscle strength and endurance. Additionally, it causes a significant decrease in dyspnea both at rest and during peak exercise. This underscores the importance of including respiratory muscle training as a supplemental approach to general conditioning exercises, especially in cases of initial respiratory muscle weakness [37]. In a controlled study of adults with idiopathic pulmonary fibrosis, Jastrzębski et al. [6] incorporated inspiratory muscle exercises using the Threshold IMP apparatus into the patients’ breathing routines. The addition of this intervention to the pulmonary rehabilitation program led to a significant decrease in dyspnea, both at rest and during exercise tests. It also resulted in a significant improvement in patients’ quality of life and exercise capacity [6].

It is known that a decline in MIP and MEP correlates with increased mortality and morbidity in older individuals. Furthermore, the activity of the diaphragm, which is the primary inspiratory muscle, may influence the symptomatic manifestation of chronic disease. Stimulating the limbic system impacts the motivation system, which is crucial for maintaining the willingness of the elderly population to engage in consistent breathing training. The deep and rhythmic nasal breaths used in inspiratory muscle training have been shown to produce rhythmic oscillations that reach the limbic area, which is responsible for emotional processing [38]. In a meta-analysis conducted by Seixas et al. [39] on inspiratory muscle training (IMT) for individuals over 60 years of age, it was demonstrated that IMT of mild to moderate intensity, undertaken between five and seven times per week for a minimum of 4 weeks, can enhance the strength of inspiratory muscles and the thickness of the diaphragm in older adults.

The limitations of our study include the absence of a control group that would perform the same pulmonary rehabilitation program above ground and the lack of a follow-up study to assess the long-term effects of the pulmonary rehabilitation. Furthermore, while maximum inspiratory pressure (MIP), maximum expiratory pressure (MEP), and sniff nasal inspiratory pressure (SNIP) are non-invasive and volitional tests, it is challenging to discern which specific respiratory muscles are weakened during their performance—whether it is the diaphragm, which actively participates in posture stabilization and body balance, or the inspiratory intercostals, scalenes, or accessory muscles such as the sternocleidomastoid. Nevertheless, this is the first study to assess respiratory muscle strength in elderly individuals with chronic respiratory diseases who underwent treatment in underground salt chambers where pulmonary rehabilitation was conducted. 

## 5. Conclusions

Speleotherapy, when combined with pulmonary rehabilitation conducted at the “Wieliczka” Salt Mine Health Resort, has been shown to enhance respiratory muscle function in older patients with chronic respiratory diseases. This improvement, measured using the MicroRPM device to assess respiratory pressures, was reflected in the increased maximal inspiratory pressure (MIP) and sniff nasal inspiratory pressure (SNIP), but was particularly evident in the maximum expiratory pressure (MEP). As a result of these findings, we suggest that incorporating increased inspiratory muscle training (IMT) into rehabilitation programs, with careful attention to postural alignment and control to activate the diaphragmatic system, could be beneficial. 

## Figures and Tables

**Table 1:** Day schedule of the rehabilitation and treatment stay at the “Wieliczka” Salt Mine Health Resort in Wieliczka [18].

Time	Timetable
15–20 min	Descent to the mine using a shaft, 700 m group walk to the treatment chambers on uneven surface of miners’ route
30–60 min	Break
30 min	Endurance training: aerobic exercise with or without tools such as sensory balls, elastic bands etc.
60–90 min	Break
30 min	Breathing exercises including breath control strategies, respiratory muscles training, resistive training, chest elasticity exercises with or without tools such feathers, pipes etc.
60–90 min	Break
35 min	Strength training with or without tools/Specialized training using N.A.P. therapy, relaxation methods and others.
30–60 min	Break or educational panel run by medical staff
15–20 min	700 m group walk to the shaft and ascent to the surface on uneven surface of miners’ route

**Table 2:** Detailed patients’ characteristics.

Variable	N (%)	Mean (SD)
sex		
Men	16 (36)	
Women	28 (64)	
Respiratory disease		
Lower respiratory tract	26 (59)	
Upper respiratory tract	18 (41)	
Age (years)		68.84 (2.9)
Height (cm)		163.26 (7.9)
Body weight (kg)		75.99 (11.3)
BMI (kg/m^2^)		28.49 (3.6)

BMI—body mass index; N—number; SD—standard deviation.

**Table 3:** Values of MIP, MEP, and SNIP [cmH_2_O] before and after the PR program in underground conditions for all participants, as well as divided by upper and lower respiratory tracts.

			Mean [cmH_2_O]	SD	Difference [cmH_2_O]	*p* *
MIP	Overall	Before	86.5	35.0		
		After	95.3	32.1	8.8	0.0070
	Lower respiratory tracts	Before	87.0	38.1		
		After	92.5	34.0	5.5	0.2157
	Upper respiratory tracts	Before	85.7	31.1		
		After	99.3	29.7	13.6	0.0054
MEP	Overall	Before	57.7	24.7		
		After	64.8	23.0	7.1	0.0004
	Lower respiratory tracts	Before	62.7	27.8		
		After	69.4	24.2	6.7	0.0233
	Upper respiratory tracts	Before	50.5	17.7		
		After	58.0	19.9	7.5	0.0022
SNIP	Overall	Before	39.5	26.6		
		After	50.7	25.5	11.2	0.0039
	Lower respiratory tracts	Before	41.9	29.1		
		After	54.9	27.8	13.0	0.0082
	Upper respiratory tracts	Before	35.9	22.8		
		After	44.7	20.9	8.8	0.1859

Abbreviations: MIP—maximal inspiratory pressure; MEP—maximal expiratory pressure; SNIP—sniff nasal inspiratory pressure; SD—standard deviation. * The *p*-value calculated using the Student’s *t*-test.

**Table 4:** Values of MIP, MEP, and SNIP [cmH_2_O] before and after the PR program in underground conditions, divided by the gender of the participants.

			Mean [cmH_2_O]	SD	Difference [cmH_2_O]	*p* *
Women	MIP	Before	72.5	25.8		
		After	82.7	23.4	10.2	0.0084
	MEP	Before	49.5	23.5		
		After	55.1	20.8	5.6	0.0190
	SNIP	Before	39.5	20.4		
		After	47.0	19.8	7.5	0.0778
Men	MIP	Before	110.9	36.3		
		After	117.2	34.1	6.3	0.3004
	MEP	Before	72.0	20.2		
		After	81.7	16.0	9.7	0.0091
	SNIP	Before	39.3	35.8		
		After	57.2	32.9	17.9	0.0237

Abbreviations: MIP—maximal inspiratory pressure; MEP—maximal expiratory pressure, SNIP—sniff nasal inspiratory pressure; SD–standard deviation. * The *p*-value calculated using the Student’s *t*-test.

**Table 5:** Values of MIP, MEP, and SNIP [cmH_2_O] before and after the PR program in underground conditions, divided by age categories.

			Mean [cmH_2_O]	SD	Difference [cmH_2_O]	*p* *
Individuals aged 65–69 years	MIP	Before	89.4	38.7		
		After	98.7	33.7	9.3	0.0322
	MEP	Before	55.7	27.3		
		After	61.2	24.8	5.5	0.0247
	SNIP	Before	38.1	25.1		
		After	51.8	27.5	13.7	0.0025
Individuals aged 70 and over	MIP	Before	82.2	29.5		
		After	90.3	29.8	8.1	0.1174
	MEP	Before	60.6	20.8		
		After	69.9	19.8	9.3	0.0071
	SNIP	Before	41.4	29.2		
		After	49.1	22.8	7.7	0.2819

Abbreviations: MIP—maximal inspiratory pressure; MEP—maximal expiratory pressure, SNIP—sniff nasal inspiratory pressure; SD–standard deviation. * The *p*-value calculated using the Student’s *t*-test.

## Data Availability

The data presented in this study are available on request from the corresponding author.
